# Exploring ChatGPT's abilities in medical article writing and peer review

**DOI:** 10.3325/cmj.2024.65.93

**Published:** 2024-04

**Authors:** Gültekin Kadi, Mehmet Ali Aslaner

**Affiliations:** Department of Emergency Medicine, Gazi University Faculty of Medicine, Ankara, Turkey

## Abstract

**Aim:**

To evaluate the quality of ChatGPT-generated case reports and assess the ability of ChatGPT to peer review medical articles.

**Methods:**

This study was conducted from February to April 2023. First, ChatGPT 3.0 was used to generate 15 case reports, which were then peer-reviewed by expert human reviewers. Second, ChatGPT 4.0 was employed to peer review 15 published short articles.

**Results:**

ChatGPT was capable of generating case reports, but these reports exhibited inaccuracies, particularly when it came to referencing. The case reports received mixed ratings from peer reviewers, with 33.3% of professionals recommending rejection. The reports’ overall merit score was 4.9 ± 1.8 out of 10. The review capabilities of ChatGPT were weaker than its text generation abilities. The AI as a peer reviewer did not recognize major inconsistencies in articles that had undergone significant content changes.

**Conclusion:**

While ChatGPT demonstrated proficiency in generating case reports, there were limitations in terms of consistency and accuracy, especially in referencing.

The rapid advance of artificial intelligence (AI) technologies is positively affecting many fields of life, including health, education, and finance (1-4). AI-based natural text processing software (natural language processing [NLP]) is able to produce logical and consistent material by rapidly processing large quantities of data. This software, used for generating text with entertaining and interesting subject matter, has recently begun affecting academic publishing. The effects of AI-based text processing software are being increasingly felt in areas such as content production, expansion, and condensation. Generative Pre-trained Transformer (ChatGPT) launched by OpenAI in November 2022 is currently the most employed and investigated NLP software in this area (5).

NLP can make the process of publishing scientific research more efficient by accelerating text production and organization processes. In addition, AI-based systems may be used in areas such as rapid article screening and quality control to fast-track the article review process.

However, the use of this technology also may lead to ethical transgressions and raise concerns regarding the originality and quality of the articles (2,6-9). In particular, it may hinder the development of thinking and writing skills among students and researchers and thus restrict critical thinking. For example, it may lead to misinterpretations in situations that require genuine experience and accumulated knowledge, such as peer review process. We are aware of no studies in which articles entirely written by AI software were peer-reviewed by human experts, or in which published articles were peer-reviewed by AI. The aim of this study is to evaluate the quality of ChatGPT-generated case reports by submitting them to peer review by human experts and assess the ability of ChatGPT to peer review medical articles.

## Material and methods

**Figure 1:**
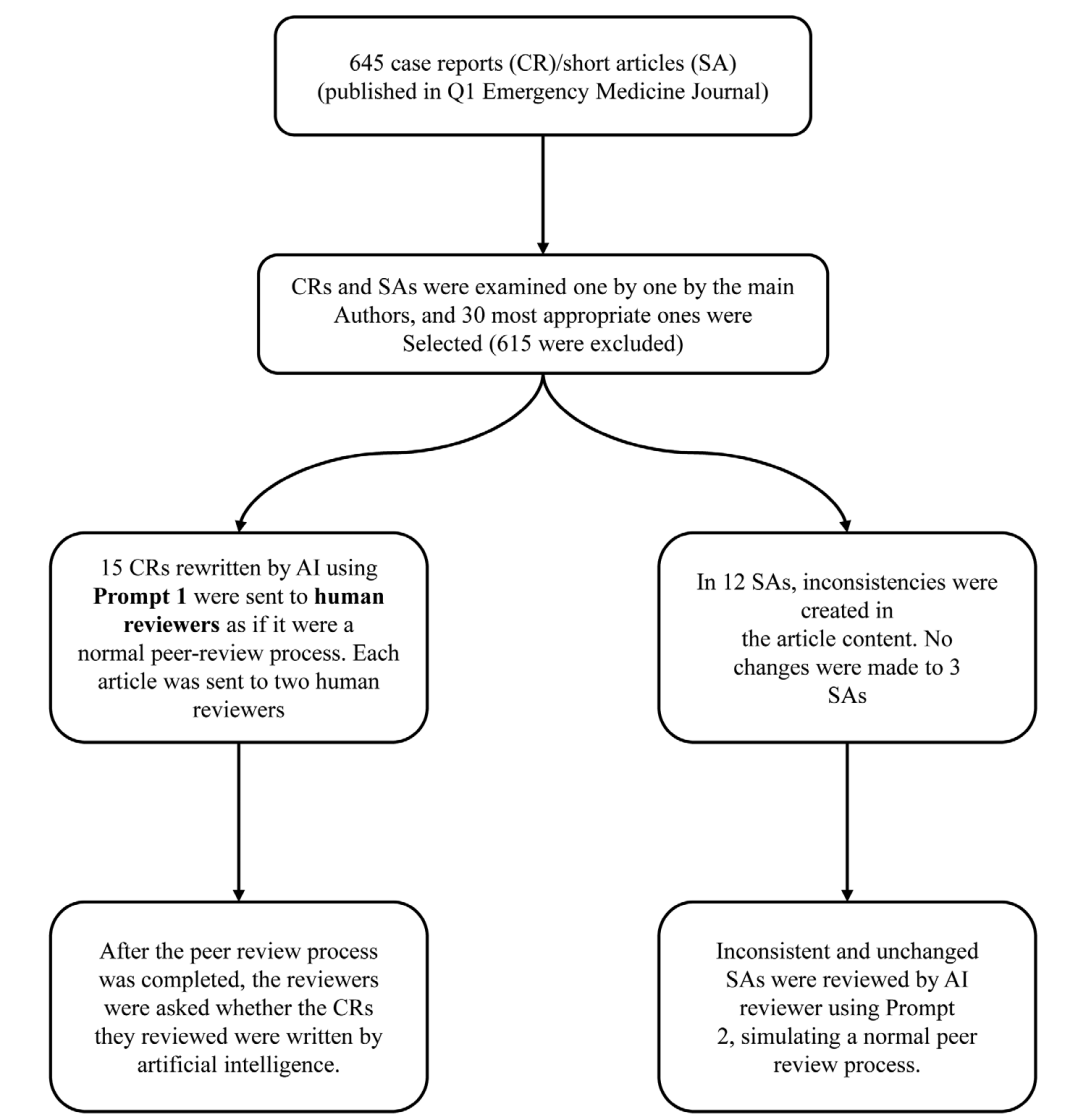
Flowchart of the study.

This study was performed between February and April 2023. In the first part of the study, ChatGPT was prompted to generate 15 case reports from scratch, which were subsequently reviewed by emergency medicine professionals. In the second part of the study, ChatGPT was asked to review 15 previously published short articles. For this purpose, we searched Q1 journals in the international Science Citation Index Expanded (SCI-E) in the field of emergency medicine for articles published between 2017 and 2020, and individually examined 645 case reports and short articles. Due to the character (approximately 2000 words for the query and 750 for the output) and visual processing limitations of ChatGPT, 15 case reports and 15 short papers with lower word counts and not including tables and figures were selected. The flowchart of the study procedure is shown in Figure 1.

For the first part of the study, only the titles and keywords of the selected case reports were collected, and the following standard query template (Prompt 1) was entered into ChatGPT 3.0 (Supplemental material 1(Supplementary Material 1)).

**Prompt 1**

Topic: “Title of original case report”

Context: A case report to be sent to an academic journal

Sections: Title, abstract, introduction, case, discussion, and references

Requirements: Include in-text citations where appropriate for at least three references from real published original articles

Language: Academic

Tone: Formal

Keywords: “from the original case report”

Total word count: At least 750 words.

When the software reached the maximum word limit and stopped writing, a simple second instruction was given: “*Continue where you left off.*” Fifteen rewritten case reports were sent for peer review to 30 emergency medicine specialists with academic experience, each report to two reviewers (Supplemental Material 2: Peer review form for case report(Supplementary Material 2)). The evaluation form and case reports were sent by email to the reviewers, who were asked to assess the text entirely in line with a normal review procedure. The reviewers were first asked about their Google Scholar h-index, years of experience in emergency medicine, and the number of manuscripts peer-reviewed last year. Then they answered specific questions about each article section with answers on a three-point scale (good, fair, and poor). They also rated the overall article in terms of originality/novelty, significance of content, quality of presentation, scientific soundness, and interest to the readers. Furthermore, they were asked to recommend a target journal for publishing of the article (low-ranking non-SCI-E, high-ranking non-SCI-E, low ranking SCI-E, high-ranking SCI-E); rate the overall merit of the article on a scale 1-10; and give the final recommendation (reject, major revision, minor revision, accept). Additional space was left for comments. The reviewers were given no information concerning whether the research involved AI. When all the reviewers had completed the forms, a second form was issued, and the participants were asked whether the reports had been written by a human or by AI software. The answers were provided on a three-point scale (human, not sure, AI).

The case reports were further evaluated by AI Text Classifier (https://platform.openai.com/ai-text-classifier) provided by OpenAI. “Possibly or likely AI-generated” results were classified as AI-generated, and “very unlikely or unlikely” results as human-generated, with the “unclear” option also being available. Finally, the case reports underwent plagiarism check (http://www.ithenticate.com).

In the second part of the study, ChatGPT was asked to review 15 previously published, short research articles. Since ChatGPT version 4.0 was launched at the end of the second part of the study, the review process continued with the new version. No change was made to three of the 15 short articles. In order to determine how the AI would respond in the event of errors, we made some changes to 12/15 articles. In two articles, the title was altered in such a way as to create an inconsistency between it and the article content; in two articles, the methodology section was partly/completely altered; in two articles, numeric data partly or entirely inconsistent with the results were created; in two articles, the references were entirely altered or removed; and in two articles, minor/major grammatical errors were created. The newly produced articles were submitted to the review process with the standard query template (Prompt 2; Supplemental material 3(Supplementary Material 3) and Supplemental material 4(Supplementary Material 4)). The study was approved by the Gazi University Ethics Committee, Ankara (2023-482).

**Prompt 2**

Review the following brief article for its suitability for publication in an academic journal and give a score of good, fair, or poor for each of the following aspects. For recommendation, give a decision, which can be reject/minor revision/major revision/accept. If the article is not rejected, give a score for the target journal, which can be a low ranking non-SCI-E journal, a high ranking non-SCI-E journal, a low ranking SCI-E journal, or a high ranking SCI-E journal.

Title

Introduction

Methodology

Results and Data analysis

Discussion

Conclusion

References

Originality / Novelty

Significance of Content

Quality of Presentation

Scientific Soundness

Interest to the readers

Ethics

Writing quality

Recommendation

Target journal (if not rejected)

“Title and main text of the manuscript”

### Statistical analysis

Continuous variables are expressed as means and standard deviations, while categorical variables are expressed as numbers and frequencies. Differences between the reviewers in terms of years of experience and opinions regarding the source of case reports were assessed with a Kruskal-Wallis test. Statistical analysis was performed with SPSS, version 22 (IBM., Armonk, NY, USA).

## Results

Thirty emergency medicine professionals peer-reviewed the case reports. The mean length of time spent by the reviewers in the field of emergency medicine was 11.4 **±** 5.6 years, and their mean h-index was 5.4 **±** 4.1. The mean number of articles reviewed by them in the previous year was 7 **±** 6.7.

### Case reports generated by ChatGPT

Each case report written by ChatGPT was evaluated by two reviewers. When individual sections were reviewed, the following percentage of reviewers rated the individual sections as good: 66.7% for titles, 33.3% for the references, 50% for the abstract, 36.4% for the introductions, 7% for the case sections, 20% for the discussion sections, and 50% for the conclusions. Examination of the references produced by ChatGPT showed that 65% were non-existent, and that 87% of the valid references were older than 10 years. The titles of these unreal references were consistent with the case reports, but when accessed using the DOI, a paper on a completely different topic appeared. The reviewers found that the majority of the references that appeared did not actually exist.

Overall, 33.3% of the reviewers rated the case reports as good in terms of quality of presentation, while 43.3% rated them as poor in terms of scientific soundness (Table 1). The mean overall merit of the case reports was 4.9 **±** 1.8 out of 10. A third of reviewers recommended rejection as a final decision, and two case reports were rejected by both reviewers. Another third recommended major revisions. In terms of the target journals suggested by the remaining 20 physicians, 11 reviewers recommended low ranking (LR) non-SCI-E, 5 recommended HR non-SCI-E, 3 recommended LR SCI-E, and 1 recommended HR SCI-E. In addition, 13.3% of the reviewers thought that the case reports had been written by AI, and 30% that they were human in origin, while 56.7% were uncertain. Only 20% of the reviewers evaluated the case reports as “of interest to the readers” and 27% rated them as good in terms of ethics.

**Table 1:** Peer review ratings of case reports generated by ChatGPT by human reviewers (N = 30)

	No. of reviewers who rated the article in terms of						
	originality /novelty	significance of content	quality of presentation	scientific soundness	interest to the readers	ethics	writing quality
Good	7	9	10	2	6	8	9
Fair	20	15	10	5	17	9	14
Poor	3	6	10	13	7	13	7

AI Text Classifier reported 93.3% of the case reports to be written by humans, while for 6.7% it reported to be “unsure.” Although the reviewers who recognized the use of AI had more years of experience and greater h-index, the differences were not significant (Table 2). The case reports’ mean plagiarism score was 17.47 **±** 7.34. Ten reviewers made additional comments regarding the case reports (Supplementary Material 5: Comments(Supplementary Material 5)). Five of these concerned incorrect references. One referee commented that the study was not appropriate to be published as it did not provide any information about patient consent.

**Table 2:** The relationship between the reviewers’ experience and opinions regarding the source of case reports

Experiences*	Human	Not sure	AI	p
Years of experience in emergency medicine	12.44 (7.601)	9.82 (3.877)	15.50 (6245)	0.284
h-index^†^	6.67 (4.610)	4.12 (2.395)	8.25 (7.365)	0.413
Peer review per year	7.44 (9002)	7.18 (5.919)	5.25 (4.717)	0.655

*Mean (standard deviation).

†Based on Google Scholar.

### Short articles reviewed by ChatGPT

In the second part of this study, ChatGPT rated three original short articles previously published in a high-impact journal in which no alterations were made as generally good. It recommended major revisions for two out of these articles, and recommended that these could be published in a journal in the LR SCI-E index. It suggested a minor revision to the third, deeming it suitable for publication in an HR non-SCI-E index.

Two articles contained titles that had been altered so as to be inconsistent with the content. ChatGPT detected no discrepancy between the title and content and evaluated the titles as good. In two other articles, the introduction section was altered so as to conflict with the text content. ChatGPT regarded these as generally good and recommended publication in HR non-SCI-E journals following minor revision. Although the software described the two articles subjected to partial and total changes in terms of methodology as generally good, it reported the methodology as fair, and recommended that these could be published in HR non-SCI-E/LR SCI-E journals after correction. Two articles contained minor and major typographical errors. The software described these articles as generally good but the writing as poor, and recommended major revision for both. The software described the two articles whose references had been altered and entirely removed as good in terms of reference evaluation and recommended publication following revision (Table 3).

**Table 3:** Peer review ratings of previously published short articles created by ChatGPT, numbers indicate the count of short articles (N = 15)

	Title	Introduction	Methodology	Results and data analysis	Discussion	Conclusion	References	Originality /novelty	Significance of content	Quality of presentation	Scientific soundness	Interest to the readers	Ethics	Writing quality
Good	13	12	7	10	14	14	14	7	14	14	10	14	15	13
Fair	2	3	8	5	1	1	1	8	1	0	5	1	0	0
Poor	0	0	0	0	0	0	0	0	0	1	0	0	0	2

## Discussion

Our results showed that with adequate prompts, ChatGPT can create appropriate introduction, case report, and discussion sections of a scientific case report. However, it also cited unreal and old sources in the references section. The review capacity of NLP was weaker than its ability to produce a written text. The software failed to detect major changes in articles in which the content was significantly altered, it was unable to fully evaluate the integrity of the text in a holistic manner, and even considered the references appropriate in an article with no references at all.

Overall, 66.6% of the reviewers evaluated the titles of the case reports produced by ChatGPT as good, and this was the report section that received the highest score. The title is an important part of an academic text as it ensures a favorable first impression and provides information about the study content (10). On the other hand, when prompting ChatGPT to generate case reports, we used the titles and key words of previously published articles. The software did not create the titles itself. It made some minor changes, but usually employed the original titles. These therefore elicited good scores from the reviewers. This is important as it confirms that the referees’ evaluations were appropriate.

The reviewers’ evaluations of the introduction section, which contains more original content, showed that the NLP produced monotonous, repetitive texts. Due to the NLP’s limited ability to produce original and up-to-date texts, the peer reviews resulted in low scores.

Overall, 67% reviewers evaluated the case reports as fair in terms of originality/novelty, 50% in terms of content, 34% in terms of quality of presentation, and 50% in terms of scientific soundness. Referee evaluations in these sections are generally powerful predictors of acceptance or refusal (11). Zhu et al reported that AI can write an in-depth discussion, and that the review article created can represent a useful electronic encyclopedia, but not useful scientific literature (12). On the other hand, Dwivedi et al emphasized that “the absence of originality” detected during their experience of article production using ChatGPT was very important in the context of creative writing, although perhaps unimportant for routine text creation functions (4). The accuracy, originality, and academic integrity of NLP in academic publishing have been described as open to debate and requiring improvement (13).

In order for articles to attract readers’ interest, they should be consistent with the literature and up-to-date, and the authors should make their language and style clearly accessible to the user (7). However, the referees in our study stated that the articles written by ChatGPT were monotonous, not up-to-date, and exhibited variations in style and a lack of fluidity. On the other hand, some authors prefer to employ AI, even grant it the status of author (14,15).

Some authors suggested that the use of AI-generated texts in academic publishing will increase the number of poor-quality or plagiarized articles, erode trust among the academic community, and lead to ethical concerns (16,17). Another ethical issue relates to the legal framework, such as determining who is to blame when the AI doctor makes a medical mistake (18).

Salvagno et al stated that human input in meta-analyses and systematic texts requiring complicated writing processes remains essential, but that ChatGPT can be used as an editing tool (19). In the present study, the referees rated the writing quality of the case reports, which require a simpler writing process, as good or fair in 77% of the cases.

The mean overall merit score for all the cases produced by NLP was 4.9 out of 10. Such a low rating explains the high rejection rate by the referees.

The mean plagiarism score in the present study was 17.47 ± 7.34. No consensus and no definite rules exist regarding the acceptable level of plagiarism in academic publications. However, a similarity figure below 15%-20% is usually acceptable to journals. Aydın et al determined a plagiarism rate of 40% in academic papers written by NLP (20,21).

In terms of responses to the question of authorship, four reviewers stated that the texts might have been produced by AI, although 17 thought they could not be sure. The reviewers who recognized AI had more years of experience and a higher h-index, but the difference was not significant, possibly due to small sample size. ChatGPT AI Text Classifier reported that 93.3% of the same case reports could have been drafted by humans. In effect, the NLP stated that almost all the texts it wrote were human in origin. The inconsistency of the results obtained from different AI detection tools reveals the inadequacy of current methods for accurately detecting scientific abstracts created by AI (9). Reviewers correctly identified 68% of abstracts created by AI (22), although these rates may change as technology advances or with the use of different AI detection software (23).

Although 4/15 articles contained obvious errors that should have led to rejection, NLP failed to detect these or report them negatively in the review. It recommended minor revision for half of these articles and major revision for the other half. It also rated the references as “good” in an article (short article 12) from which the references had been entirely removed. This indicates that ChatGPT can comment on a non-existent text section. During the entire peer review, it rated only those articles that contained typos or grammatical errors as poor. This indicates that NLP is unable to grasp the logical construction, continuity, and integrity of the texts, but does detect typographical and grammatical errors. Elsewhere in the literature, NLP software such as ChatGPT has been reported to accelerate the writing process, improve the main lines and details of a paper, and to enhance the writing style (24).

A limitation of the study is that only 15 case reports and 15 short articles were examined in the study. Increasing the sample size and using reports from different disciplines may increase the generalizability of the findings. The AI programs used are developing and updated over time. This and similar studies reveal repetition with different software.

In conclusion, we believe that with its current technological infrastructure, ChatGPT is not capable of producing original and academic text, but that it can be used for text editing. In the future, it may become necessary to use plagiarism software that can detect AI in academic publishing.
