# Supplementary material for: Exploring ChatGPT's abilities in medical article writing and peer review
Source: Croat Med J. 2024 Apr;65(2):93–100. doi: 10.3325/cmj.2024.65.93 (PMC11074943; doi:10.3325/cmj.2024.65.93)
Supplement: Supplementary material 1 [file CroatMedJ_65_s010.pdf]

Peer review for case report

Please, answer all questions.

1. How many years have you been doing emergency medicine? *

2. How many articles did you peer review last year? *

3. What is your H index on the Google academic score? *
   https://scholar.google.com/

4. Title of case report *

5. Sections *

	Good/Appropriate	Fair/Just Appropriate	Poor/Inappropriate
**Is the title informative, and comprehensive?**			
**Does the abstract represent the main content of the paper with necessary detail?**			
**Does the introduction provide sufficient background?**			
**Does the case presentation have appropriate details of the case, including demography, assessment, findings, and investigations?**			
**Does the discussion emphasize why the case is important to medicine, and have adequate literature review?**			
**Does the conclusion represent the core key message of the**			

**case?**			
**Are all the cited references correct (from actual published articles), and relevant to the research?**	⬭	⬭	⬭

6. General *

	Good/Appropriate	Fair/Just Appropriate	Poor/Inappropriate
**Originality / Novelty**	⬭	⬭	⬭
**Significance of Content**	⬭	⬭	⬭
**Quality of Presentation**	⬭	⬭	⬭
**Scientific Soundness**	⬭	⬭	⬭
**Interest to the readers**	⬭	⬭	⬭

7. Target journal *

- ⬭ Low ranking non-SCI-E journal
- ⬭ High ranking non-SCI-E journal
- ⬭ Low ranking SCI-E journal
- ⬭ High ranking SCI-E journal

8. Overall Merit *

	1	2	3	4	5	6	7	8	9	10	
Low	◯	◯	◯	◯	◯	◯	◯	◯	◯	◯	Highest

9. Recommendation *

- ◯ Reject
- ◯ Minor revision
- ◯ Major revision
- ◯ Accept

10. Additional comments
