# Supplementary material for: Exploring ChatGPT's abilities in medical article writing and peer review
Source: Croat Med J. 2024 Apr;65(2):93–100. doi: 10.3325/cmj.2024.65.93 (PMC11074943; doi:10.3325/cmj.2024.65.93)
Supplement: Supplementary material 2 [file CroatMedJ_65_s011.pdf]

Comments of Reviewers

1. The cited references are mostly old (more than 10 years), except references 9 and 10. These 2 references are somehow pretty relevant, and can not be found in the literature.
2. Dear editor, Thank you for giving me the opportunity to evaluate the work. Central venous catheterization is one of the most frequently applied interventions in Emergency Medicine residency training and afterwards. Although this intervention can be performed more easily, especially with the increase in the use of USG, and the possibility of complications is reduced, it can result in serious adverse events, although rare. Thank you to the authors for this interesting case report. However, although the title of the article is remarkable, the presentation is sloppy, it is not fluent, there are too many repetitions, and it is not appropriate for this study to be published due to reasons such as not sharing any information about patient consent, not reporting the references correctly, etc. Kind regards.
3. This is a well-written case report, but there is a significant problem with the references. I cannot find one of the references (third reference "Chawla R, Sood A, Mittal G. Hashimoto's encephalopathy with urinary retention: a rare presentation. J Neurosci Rural Pract. 2016;7(2):280-2." in Google, PubMed and the journal's original archives. This is a major problem; even though the case report is well-written and the case is infrequent, it is completely unacceptable. I strongly advise rejecting this case report. Nevertheless, while reading the case report, I cannot stop asking myself what made the authors check the anti-thyroid antibodies but not perform a lumbar puncture or spinal radiologic evaluation.
4. A single case report cannot provide a sufficient level of evidence about the effectiveness of a drug in a specific diagnosis. Randomized controlled studies are needed in this regard. These studies can also be done after obtaining mandatory and strong ethical approval.
5. Why did you suspect hypothyroidism in that patient? Do you usually study tests for hypothyroidism when you see a swollen leg in your emergency practice? What led you to suspect the diagnosis? The clinical aspect should be detailed. Additionally, the mechanism that is responsible for the development of compartment syndrome in hypotiroidism should be detailed. Regards.
6. I think this scientific article is important for healthcare providers instead of public awareness. The authors should explain the electrolyte abnormalities and why they did not use DigiFab. Kind regards.
7. There is an example of a similar case in the literature. More demographic data on snake bites should also be presented in the introduction section (regional differences etc.) In the case report, no information is given about the vital signs of the patient. Clinical findings suggestive of cholinergic toxidroma should also be presented. It should be detailed. Only one of the references given was accessed and other references were not found. This is considered a major mistake by the author.
8. The literature contribution level is low. There are similar studies in the literature. I don't think it's acceptable.
9. A connection could not be established between the information given during the patient's presentation. The relationship between the patient's complaint of urinary

retention and Hashimoto's thyroiditis is not clear. Finally, the third reference could not be accessed from the archive of the journal and other databases.

10. Congratulations to the authors on this work. The work was written in an appropriate pattern and style and reached its goal. However, this case report is still a well-known issue in the literature. In the literature, many cases of trauma-related or non-traumatic compartments in patients with hypothyroidism have been presented. There are even cases with four extremity compartments. Therefore, the contribution of this case to the current literature is limited. In addition, in the case report section, it is not clear how long the patient stayed in the hospital, whether it was complicated or not, which treatment, which dose and how much.. Thank you

11. This case describes the serious health problems and death of a woman who consumed an herbal weight loss supplement purchased online. This situation highlights the unregulated nature of herbal supplements and the presence of potentially harmful substances they contain. However, the presentation is inadequate because there is no detailed information about the supplement. There is a lack of information on how the herbal supplement was manufactured, what ingredients it contains, and in what dosage it should be taken. Therefore, while the presentation can serve as a general warning that herbal supplements are not safe, it cannot be used as a definitive recommendation for an individual supplement. In addition, the detection of the mentioned dietary supplement by blood tests will establish a direct link between the current clinic and this dietary supplement. The authors must explain if this test was performed and if not, why it was not performed. If more information were provided, a more comprehensive analysis of the case could be made. Factors such as the duration of supplement use, the dose used, and the patient's general health could be examined. In this way, a more accurate understanding of when herbal supplements are safe and when they might be harmful could be gained. In summary, this case presentation may serve as a warning about the unregulated nature of herbal supplements. However, more information is needed for a more comprehensive analysis.
