# Supplementary figures and images for: Exploring ChatGPT's abilities in medical article writing and peer review
Source: Croat Med J. 2024 Apr;65(2):93–100. doi: 10.3325/cmj.2024.65.93 (PMC11074943; doi:10.3325/cmj.2024.65.93)

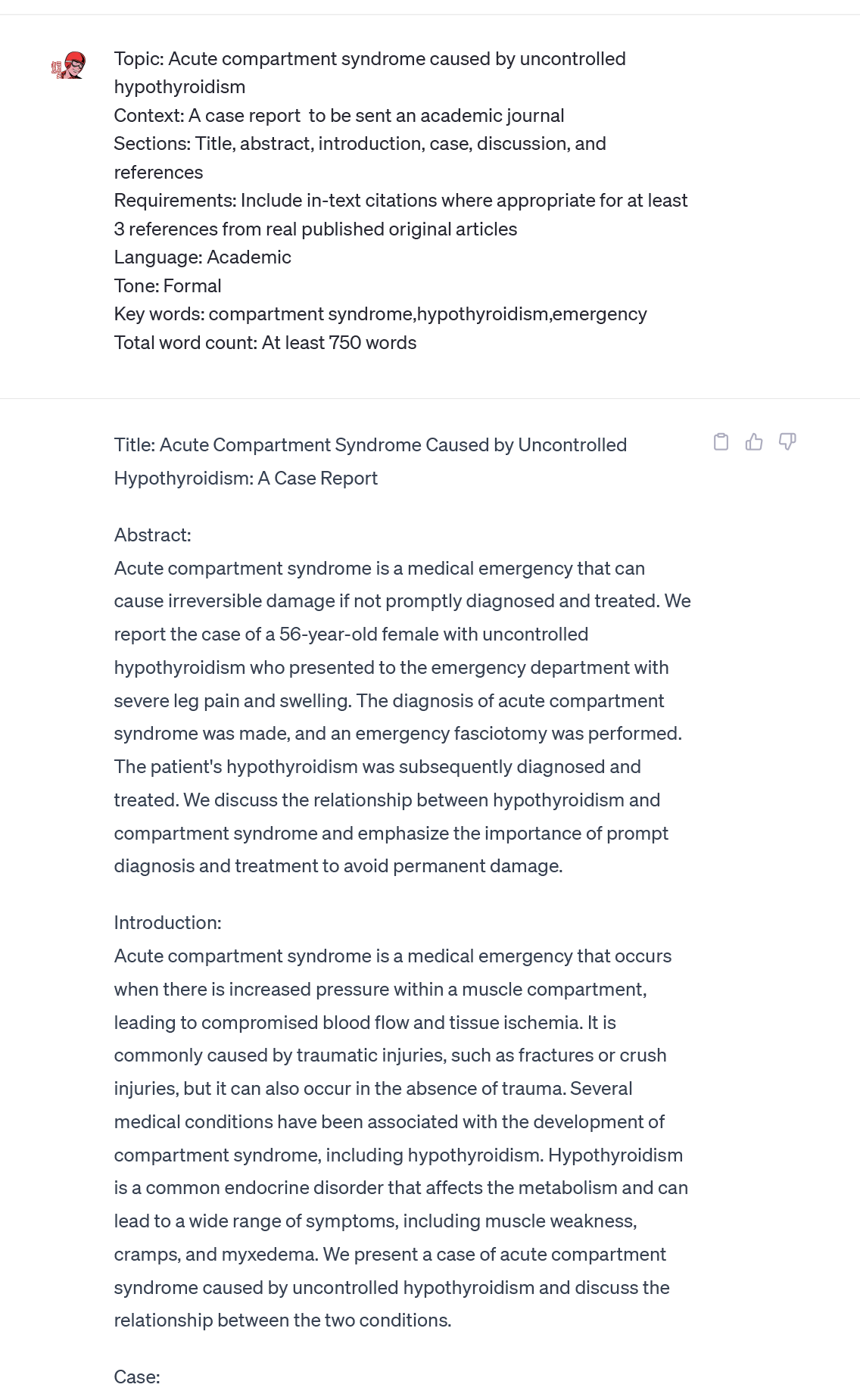

Supplement: Supplementary material 3 [file CroatMedJ_65_s012.tif]

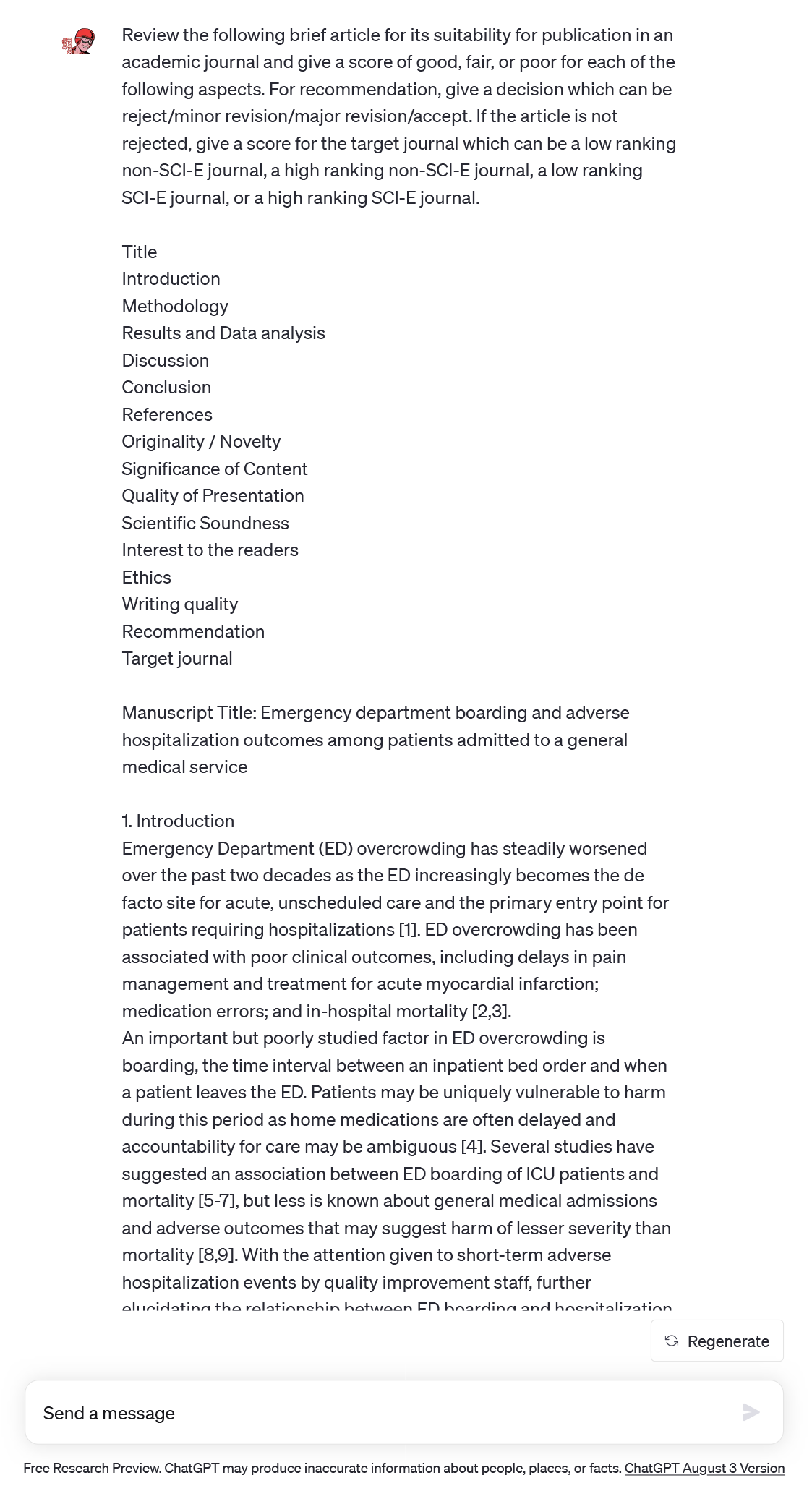

Supplement: Supplementary material 4 [file CroatMedJ_65_s013.tif]

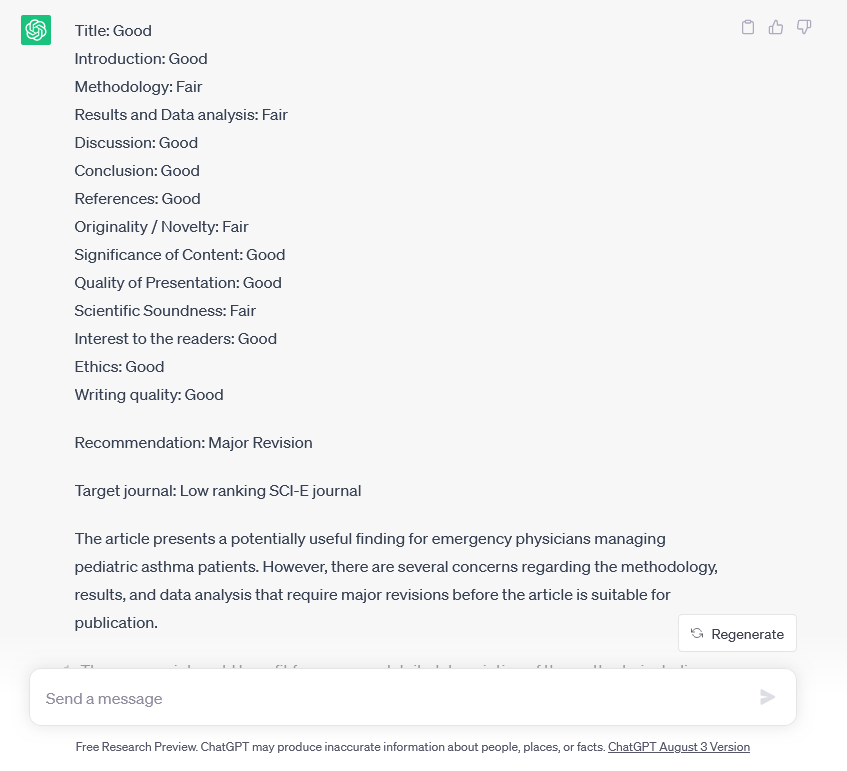

Supplement: Supplementary material 5 [file CroatMedJ_65_s014.tif]
